# Character Strengths Are Related to Students’ Achievement, Flow Experiences, and Enjoyment in Teacher-Centered Learning, Individual, and Group Work Beyond Cognitive Ability

**DOI:** 10.3389/fpsyg.2020.01324

**Published:** 2020-07-16

**Authors:** Lisa Wagner, Mathias Holenstein, Hannah Wepf, Willibald Ruch

**Affiliations:** ^1^Department of Psychology, University of Zurich, Zurich, Switzerland; ^2^Department of Psychology, University of Basel, Basel, Switzerland

**Keywords:** character strengths, socio-emotional skills, positive education, optimal experience, trait activation theory

## Abstract

While character strengths have been found to predict educational outcomes beyond broad personality traits and cognitive ability, little is known about their differential contribution to success and positive learning experiences in different school settings. In this study, we use trait activation theory to investigate the relationships of students’ character strengths with achievement, flow experiences, and enjoyment in different learning situations (i.e., teacher-centered learning, individual tasks, and group work). In studying these relationships, we controlled for psychometric intelligence. Secondary school students (*N* = 255; 46.3% male; mean age = 14.5 years) completed a self-report measure of character strengths, the VIA-Youth (Park and Peterson, 2006b). Cognitive ability was assessed using a standardized intelligence test (PSB-R; Horn et al., 2003) at baseline. Three months later, students completed the Flow Short Scale (Rheinberg et al., 2003) adapted to the three learning situations and indicated their typical enjoyment of these situations. Both the students and their teachers (*N* = 18; 50% male; mean age = 44.8 years) provided ratings on school achievement in each of the three learning situations. Results indicate that, as expected, (a) certain character strengths (love of learning and perseverance) show consistent relationships with achievement and positive learning experiences (flow and enjoyment) above and beyond cognitive ability across all learning situations, whereas (b) other character strengths show differential trait-outcome relationships (e.g., the character strength of teamwork was predictive of achievement and positive learning experiences in group work). Taken together, these results suggest that different character strengths play a role in different school situations and that their contribution to explaining variance in educational outcomes is incremental to the contribution of cognitive ability.

## Introduction

As early as 1940, non-cognitive variables were discussed as important predictors of educational outcomes that could add to the predictive value of cognitive ability (Harris, 1940). Many decades later, there is substantial evidence that personality traits explain variance in educational outcomes (Poropat, 2009) and also do so incrementally above the influence of cognitive ability (e.g., Lechner et al., 2017). However, much is still unknown about which aspects of students’ learning experiences and performance are influenced by individual differences in cognitive and non-cognitive (i.e., personality) traits and the most useful level of analysis (i.e., broader vs. narrower traits; see O’Connor and Paunonen, 2007).

In the present study, we use the concept of character strengths (Peterson and Seligman, 2004) to investigate the role of a comprehensive set of (narrower) positively valued personality traits. While previous studies found character strengths to go along with overall school achievement (e.g., Wagner and Ruch, 2015), when controlling for broader personality traits and cognitive ability (Wagner and Ruch, 2020), school does not represent a uniform situation but rather a range of different settings, in which achievement and positive learning experiences might be facilitated by different personality traits. Therefore, we aimed at studying whether character strengths explain variance in achievement across different learning situations – namely teacher-centered learning, individual tasks, and group work – above and beyond cognitive ability. Given the relevance of positive learning experiences both for overall well-being (e.g., Stiglbauer et al., 2013) and for future achievement (e.g., Engeser and Rheinberg, 2008), we also include variables related to well-being by studying the relationships of character strengths to the experience of flow and enjoyment in the different learning situations.

### Character Strengths

Building on the theoretical framework of the Values in Action (VIA) classification (Peterson and Seligman, 2004), character is defined as a set of positive characteristics shown in feelings, thoughts, and actions. The VIA classification suggests a hierarchical structure of character where 24 character strengths are organized under six broad virtues: (1) wisdom and knowledge (encompassing the character strengths of creativity, curiosity, judgment, love of learning, and perspective), (2) courage (i.e., bravery, perseverance, honesty, and zest), (3) humanity (i.e., love, kindness, social intelligence), (4) justice (i.e., teamwork, fairness, and leadership), (5) temperance (i.e., forgiveness, humility, prudence, and self-regulation), and (6) transcendence (i.e., appreciation of beauty and excellence, gratitude, hope, humor, and spirituality). In that sense, character strengths are the “psychological processes or mechanisms that define the virtues” (Peterson and Seligman, 2004, p. 13). By definition, character strengths are ubiquitous, positively morally valued, fulfilling, trait-like, distinct, and measurable individual differences that contribute to optimal development across the lifespan (Peterson and Seligman, 2004). Importantly, character strengths are defined as malleable, which makes them ideal targets for interventions (for an overview in the educational context, see Lavy, 2019).

Character strengths also seem to be measurable and relevant in young people. Previous research has established that character strengths are already present in young children (Park and Peterson, 2006a) and can be reliably and validly measured using self-reports from the age of 10 years (e.g., Park and Peterson, 2006b; Ruch et al., 2014). A number of studies using those instruments established robust associations between character strengths and well-being among adolescents across different cultures (e.g., van Eeden et al., 2008; Gillham et al., 2011; Toner et al., 2012; Ruch et al., 2014).

### Character Strengths and Educational Outcomes

How do character strengths relate to educational outcomes? Evidence suggests that the character strengths of love of learning and perseverance are particularly conducive to a range of educational outcomes (e.g., Weber and Ruch, 2012; Shoshani and Slone, 2013; Wagner and Ruch, 2015, 2020; Weber et al., 2016). However, previous studies suggest that, depending on the outcomes assessed (e.g., school achievement, school satisfaction, or positive relationships at school), different character strengths are additionally of relevance. For instance, the character strengths of zest and social intelligence are relevant in explaining variance in positive affect at school, whereas the character strengths of teamwork, hope, self-regulation, and love are most strongly associated with low negative affect at school (Weber et al., 2016). Specifically, the strengths found to be associated with achievement and with positive experiences at school overlap strongly, but some strengths (such as prudence) tend to show stronger relationships with achievement and other strengths (such as zest) tend to show stronger relationships with positive experiences at school. Recently, it was also demonstrated that a number of character strengths still predicted a range of educational outcomes when cognitive ability and personality traits of the five-factor model were controlled for (Wagner and Ruch, 2020).

### Differential Relationships Between Personality or Character and Educational Outcomes

Studies on the relationships between character strengths and achievement almost exclusively rely on overall school achievement, or GPA. However, a first hint for differential relationships is represented by the finding that character strengths are generally more strongly related to grades in core academic subjects than to grades in non-academic subjects (e.g., physical education, and arts; Wagner and Ruch, 2015). Academic achievement is not a unidimensional construct and therefore, using overall school achievement or only using school grades as criterion might not allow for uncovering relationships with specific components of achievement (see O’Connor and Paunonen, 2007; Poropat, 2009). This idea is supported by findings that demonstrate differential trait-outcome relationships of the personality dimensions of the five-factor model for different school subjects or different assessments of educational achievement (e.g., Spengler et al., 2013; Zhang and Ziegler, 2016; Brandt et al., 2020). This underlines the need for a more fine-grained examination of the associations between personality traits and educational outcomes. Using broader and more varied criterion measures of academic performance than GPA to study their relationships with personality traits (e.g., Kappe and van der Flier, 2010) has generally yielded two conclusions: First, certain traits (mainly conscientiousness) are consistently positively related with academic performance irrespective of the chosen measure. Second, for a number of personality traits (such as extraversion or neuroticism), the existence and size of relationships with academic achievement depend on how achievement is measured (i.e., GPA, thesis, performance in a group project, etc.).

In interpreting such findings and in hypothesizing relationships between character strengths and educational outcomes, we relied on the theoretical framework of trait activation theory (Tett and Guterman, 2000; Tett and Burnett, 2003). The theory’s central premise is that situations differ in their relevance to any given trait, which is a well-accepted idea (see, e.g., Allport, 1937). A second premise of the theory assumes that trait expression is a rewarding experience – that is, individuals enjoy situations that allow the expression of their traits (Tett and Burnett, 2003). Trait expression (i.e., showing trait-related behavior) in a given situation is enabled by a set of situational cues, which can also be construed as opportunities or expectations. While much work on trait activation theory refers to predicting work-related outcomes, these ideas can also be applied to predicting educational outcomes (see Brandt et al., 2020). Brandt et al. (2020) argue that, for instance, different ability-grouped school tracks represent different learning contexts with distinguishable characteristics. These characteristics include different instructional styles as well as behavioral norms and expectations. Based on the notions of trait activation theory, these serve as situational cues that activate different sets of traits, which in turn causes differences in trait-performance associations between academically oriented and vocationally oriented school tracks. Specifically, Brandt et al. (2020) found, in a large sample of German students in grade nine, that conscientiousness had a stronger positive association with school performance in academic than in vocational school tracks. This finding supports the hypothesis that conscientiousness is activated to a stronger degree in a setting with higher academic demands.

### The Role of Learning Situations as Trait-Relevant Learning Contexts

Trait activation theory (Tett and Guterman, 2000; Tett and Burnett, 2003) assumes that traits are activated in response to cues within the situation. In the educational context, these cues can be located within (a) the task a student performs, (b) the social environment a student is in, or (c) the wider organizational context (Brandt et al., 2020). Differential trait-performance relationships have been observed across different types of performance assessments, such as grades or performance in standardized tests (which might mostly represent a variation within the task), grades in various subjects (again mostly a variation within the tasks), and different ability-grouped school tracks (a variation at the organizational level). Up to now, little attention has been paid to the second aspect, the students’ social environment. Yet, different learning situations that teachers use in organizing their school lessons (see Rubin and Hebert, 1998; Rimm-Kaufman et al., 2005; Meyer, 2013) may be an important cause of variability. Diverse learning situations (e.g., teacher-centered learning, individual tasks, or group work) are likely to impose differential expectations and norms for students’ behavior, thus activating traits differentially, which results in differential trait-performance associations.

Learning situations can be described as either teacher-centered learning or student-centered learning (e.g., Rubin and Hebert, 1998; Rimm-Kaufman et al., 2005), with the latter including both individual tasks and group work. *Teacher-centered learning* is characterized by the leading role of the teacher in presenting the lessons’ contents, either in a lecture-type presentation or through a moderated conversation in class. When working on *individual tasks*, students are independently working on assignments. *Group work* is characterized by students working together on assignments in (small) groups (see Meyer, 2013). A varying social environment characterizes these different learning situations: Teacher-centered learning typically involves mainly interactions with the teacher, with the entire classroom present. Individual work features minimal interactions with others and a single focus on the task given. In contrast, group work is characterized by a lot of interactions with peers and a need for cooperation.

### Aims of the Present Study and Hypotheses

The present study aims at investigating whether students’ character strengths predict both achievement and positive learning experiences (flow experiences and enjoyment) in different learning situations (i.e., teacher-centered learning, individual tasks, and group work) over and above cognitive ability. Drawing on trait activation theory, we assume that character strengths (as trait-like individual characteristics) are expressed in response to trait-relevant situational cues, thus giving rise to behaviors that impact performance and the level of achievement in this situation, and that their expression leads to positive learning experiences. As a consequence, we expect different character strengths to be related to positive learning experiences and achievement in different situations.

**FIGURE 1:**
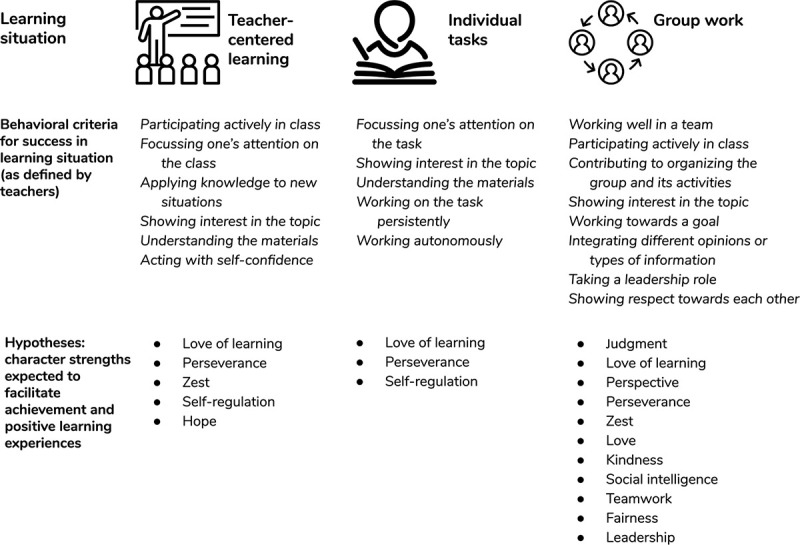
Teachers’ definitions of behaviors related to success in learning situations and hypothesized relationships of character strengths with achievement and positive learning experiences. Teachers’ answers were content-coded and are listed when they were mentioned at least three times (i.e., by at least 16.7% of the teachers).

We derived a set of hypotheses regarding specific character strengths and achievement and positive learning experiences in different learning situations based on several sources: (a) theoretical assumptions on character strengths (Peterson and Seligman, 2004) and characteristics of the three learning situations studied (see Meyer, 2013), (b) trait activation theory (Tett and Guterman, 2000; Tett and Burnett, 2003), (c) previous findings on the relationships between character strengths and school achievement (e.g., Weber and Ruch, 2012, 2015; Weber et al., 2016) and differential personality-outcome associations (e.g., Kappe and van der Flier, 2010), and (d) teachers’ definitions of achievement in the three learning situations. To obtain these definitions, we asked participating teachers (*N* = 18) to provide their own definitions of achievement (i.e., what it means to be successful and to show a good performance in each of the learning situations) using an open-ended format at the first measurement occasion (i.e., 3 months before the outcomes variables were assessed). The answers were content-coded and the most common behavioral criteria for achievement that were mentioned are summarized in Figure 1.

We hypothesized that some character strengths (in particular, love of learning and perseverance) should be conducive to academic achievement and positive learning experiences across a wide range of settings, whereas other strengths should specifically contribute to achievement and positive learning experiences in certain settings as they are specifically activated by cues present in these contexts. Specifically, we expected that love of learning and perseverance would be conducive to achievement *across all learning situations*. This was also supported by the fact that teachers mentioned behaviors that are expressive of the character strengths of love of learning (e.g., “showing interest in the topic”) and perseverance (e.g., “working on the task persistently” and “working toward a goal”) as relevant for achievement across all learning situations (see Figure 1).

Achievement in *teacher-centered learning* was hypothesized to be additionally related to specific character strengths since it requires active participation in class (zest), the ability to focus one’s attention (self-regulation), and self-confidence (hope). As working on *individual tasks* requires working in a self-regulated manner, we also expected achievement in individual tasks to be related to the strength of self-regulation. Successfully working on a task in a group also requires integrating different opinions or types of information (strengths of judgment and perspective) and working well with other students (strengths of love, kindness, social intelligence, teamwork, fairness, and leadership), which is why we assumed that these strengths would be associated with better performance in *group work*. We expected those strengths that should go along with better performance to also relate to positive learning experiences (flow and enjoyment) in the respective situation. With regard to flow experiences, we additionally expected that creativity, curiosity, judgment, love of learning, perseverance, zest, self-regulation, and hope would be conducive to experiencing flow in all of the learning situations, in line with earlier findings (Wagner and Ruch, 2020). Figure 1 gives an overview of the hypothesized relationships.

## Materials and Methods

### Participants

We calculated the required sample size using G^∗^Power 3.1 (Faul et al., 2009) based on a power of at least 0.80 to detect an effect of *r* = 0.20 (based on previous studies’ results; e.g., Wagner and Ruch, 2015) using an α-level of 0.01 and one-tailed tests. This resulted in a required sample size of at least *N* = 247.

Altogether, we collected data of 301 participants in 19 classrooms. Data of 48 participants were excluded from the analyses because they had missing data in several relevant instruments (*n* = 18, mostly because they did not participate in both data collections), did not complete the intelligence test (*n* = 14, i.e., one classroom), showed response patterns indicative of careless responding (*n* = 8, determined by examining repeated answers, the consistency of recoded and non-recoded items, and response times), or had too little knowledge of German (*n* = 6). Thus, the analyzed sample consisted of *N* = 255 students (46.3% boys and 53.7% girls) from 18 different classrooms. At the time of the first data collection, participants had a mean age of 14.49 years (*SD* = 1.07; ranging from 12.42 to 18.75 years). Most (83.2%) were between 13 and 15 years old. In Switzerland, secondary schools can be categorized into two tracks: Around one-quarter of participants attended schools with basic requirements (i.e., with a vocational orientation) and 76.5% of participants attended schools with augmented requirements (i.e., with an academic orientation), which approximately represents the distribution of schools in the respective communities.

The sample of teachers consisted of *N* = 18 teachers (8 female and 10 male) with a mean age of 43.67 years (*SD* = 12.16, ranging from 24 to 60 years). They had been working as teachers for on average of 19.17 years (*SD* = 12.46). In the Swiss secondary school system, students in one classroom typically attend most classes together as a group. The teachers participating in the present study were their homeroom teachers in most cases (i.e., in 94.4%) and typically taught several school subjects to the same class (on average 10.78 hours per week, with *SD* = 4.08). All teachers had also been teaching the respective students for at least 6 months (*M* = 15.44 months, *SD* = 9.79). Thus, it can be assumed that they were sufficiently familiar with the students to rate their achievement in different learning situations.

### Instruments

To assess students’ character strengths, we used the *Values in Action Inventory of Strengths for Youth* (VIA-Youth; Park and Peterson, 2006b) adapted to German by Ruch et al. (2014), which is based on the VIA classification (Peterson and Seligman, 2004) and consists of 198 items with a 5-point answer format (from 5 = *very much like me* to 1 = *not like me at all*). A sample item is “I don’t boast about what I achieve” (character strength of humility). The VIA-Youth has demonstrated its reliability and validity in a number of studies (e.g., Park and Peterson, 2006b; Ruch et al., 2014). In this study, the internal consistency coefficients of the 24 scales yielded a median of α = 0.77 (ranging from 0.67 to 0.88, see Table 1). As not all VIA-Youth scales can be assumed to be fully unidimensional, these coefficients might be biased and need to be interpreted with caution. However, previous research (Ruch et al., 2014) testing other forms of reliability, namely test-retest correlations across 4 months (median *r*_*tt*_ = 0.72), provides further evidence for the reliability of the measure.

**TABLE 1:** Descriptive statistics, internal consistencies, and correlations with age, gender, and school track for VIA-Youth scales.

	*M*	*SD*	*Min*	*Max*	α	*r*_*age*_	*r*_*gender*_	*r*_*track*_
Creativity	3.60	0.62	1.50	5.00	0.77	−0.13*	–0.10	0.02
Curiosity	3.54	0.58	2.00	5.00	0.76	–0.06	–0.10	0.09
Judgment	3.52	0.54	2.25	5.00	0.73	–0.02	0.04	–0.01
Love of learning	3.44	0.59	1.63	4.88	0.75	–0.08	0.21*	0.02
Perspective	3.68	0.49	2.38	4.88	0.70	–0.03	0.17*	0.12
Bravery	3.73	0.58	2.38	5.00	0.79	–0.03	0.10	0.00
Perseverance	3.49	0.60	1.56	5.00	0.79	–0.10	0.23*	−0.16*
Honesty	3.78	0.57	1.25	5.00	0.82	–0.01	0.27*	–0.04
Zest	3.52	0.56	1.88	5.00	0.73	−0.18*	0.04	–0.03
Love	4.04	0.63	1.89	5.00	0.81	–0.03	0.21*	–0.06
Kindness	4.08	0.55	2.11	5.00	0.82	–0.10	0.41*	–0.02
Social intelligence	3.78	0.48	2.25	5.00	0.67	0.02	0.19*	0.09
Teamwork	3.99	0.49	2.13	5.00	0.72	0.01	0.23*	0.07
Fairness	3.58	0.55	1.89	4.89	0.72	0.03	0.32*	0.08
Leadership	3.34	0.67	1.25	5.00	0.85	0.01	0.01	0.01
Forgiveness	3.78	0.62	1.29	5.00	0.77	0.04	0.03	0.15
Humility	3.69	0.57	1.67	5.00	0.73	–0.02	0.25*	0.09
Prudence	3.34	0.58	1.63	4.63	0.73	0.04	0.15*	0.03
Self-regulation	3.49	0.59	1.56	5.00	0.75	0.06	0.16*	0.00
Beauty	3.51	0.69	1.63	5.00	0.79	0.01	0.37*	0.14*
Gratitude	4.18	0.53	2.00	5.00	0.79	–0.03	0.17*	–0.08
Hope	3.80	0.59	1.75	5.00	0.80	0.02	–0.03	0.02
Humor	3.96	0.60	1.67	5.00	0.79	–0.01	–0.10	0.10
Spirituality	3.51	0.99	1.00	5.00	0.88	−0.17*	0.12	−0.21*

N = 255. Beauty = Appreciation of beauty and excellence. Age: 12.42–18.75 years. Gender: 0 = male, 1 = female. Track: 0 = school with vocational orientation, 1 = school with academic orientation. * p < 0.05 (two-tailed).

To assess school achievement across the different learning situations, we used both *teacher- and self-reports*. For each learning situation, teachers were asked to rate each student on two items (e.g., for individual tasks “The student is successful in individual tasks.” and “The student performs well in individual tasks.”) using a 7-point scale (ranging from 1 = *completely disagree* to 7 = *completely agree*). Each learning situations was explained in a short description (provided in Supplementary Material). For example, individual tasks were introduced by the following description: “At school, there are situations, in which the teacher gives the students a task to complete. In some of these situations, students are asked to work on these tasks individually. We refer to these situations as ‘individual tasks.”’ Since the two items correlated highly [*r*(253) = 0.86 for teacher-centered learning, *r*(253) = 0.93 for individual tasks, and *r*(253) = 0.93 for working in groups, all *p* < 0.001], we used the means across the respective two items in our analyses. Similarly, students were also provided with descriptions of the learning situations (see Supplementary Material) and asked to rate their achievement in each learning situation (e.g., for individual tasks “I am successful in individual tasks.” and “I perform well in individual tasks.”) using a 7-point scale (ranging from 1 = *completely disagree* to 7 = *completely agree*). Again, the two respective items correlated highly [*r*(253) = 0.75 for teacher-centered learning, *r*(253) = 0.87 for individual tasks, and *r*(253) = 0.78 for working in groups, all *p* < 0.001], so we also used the means in the analyses.

To assess habitual flow experiences across the different learning situations, we used an adaptation of the Flow Short Scale (FSS; Rheinberg et al., 2003). The FSS consists of 10 items (answered on a 7-point scale) covering different components of flow experiences and was designed to assess flow in specific situations. We adapted the scale to assess habitual experiences by presenting it with an instruction to think of the different learning situations (referring to the same description as for the achievement rating). The three versions of the scale (and a version assessing experiences in school in general, which is not relevant for the present study) were presented in a randomized order to avoid systematic order effects. In the present study, these three scales reached internal consistencies of α = 0.82 (teacher-centered learning), α = 0.89 (individual tasks), and α = 0.86 (group work).

To assess the enjoyment of learning situations, we used three items, one for each situation (e.g., for individual tasks “I enjoy individual tasks.”). Students rated to what extent they agreed with each statement on a 7-point scale (ranging from 1 = *completely disagree* to 7 = *completely agree*).

To assess psychometric intelligence, we used the *Prüfsystem für Schul-und Bildungsberatung für 6. bis 13. Klassen, Revidierte Fassung* (Testing System for Scholastic and Educational Counseling, Grades 6–13 –revised version; PSB-R 6–13; Horn et al., 2003). The PSB-R 6–13 was designed for use in educational settings and encompasses the assessment of reasoning and verbal intelligence (including school-specific knowledge) as well as concentration. It consists of nine subtests (three for the assessment of verbal intelligence, four for the assessment of reasoning, and two for the assessment of concentration). The PSB-R 6–13 has previously demonstrated strong convergent validity with other measures of cognitive ability as well as criterion validity in the prediction of outcomes such as school grades (Horn et al., 2003). In the present study, we used the total score, which is based on all nine subtests and offers a comprehensive measure of cognitive ability that was found of particular relevance to predicting school achievement. For the analyses, we used age-standardized scores (*M* = 100; *SD* = 10) of this total score.

### Procedure

The study’s procedures were approved by the institutional ethical board at the Faculty of Philosophy at the University of Zurich. All participants gave their written consent and participated voluntarily. Students under the age of 14 years were provided written permission to participate by a parent or legal guardian. As an incentive, participating students were offered individualized feedback on their character strengths.

Data presented here were collected as part of a larger project and the sample presented here overlaps (by 70.6%) with Wagner and Ruch (2020). Wagner and Ruch (2020) studied the incremental validity of character strengths in predicting educational outcomes beyond intelligence and the personality traits of the five-factor model. Two of the predictors overlap between both studies, but none of the outcomes. Specifically, Wagner and Ruch (2020) focused on educational outcomes in general, whereas the present study investigates differential trait-outcome associations across different learning situations. Questionnaire data were collected on school computer or tablets, whereas the intelligence test was administered in paper/pencil-format. The VIA-Youth and the intelligence test (PSB-R 6–13) were completed at a baseline assessment, and the data on outcome variables (achievement ratings by teachers and students, FSS, and enjoyment ratings) were collected about 3 months later (*M* = 95.49 days, *SD* = 3.87, range: 84–102). Both data collections also contained other measures not relevant to the present study.

### Data Analysis

To account for the nested structure of the data, we first computed ICC(1) coefficients to evaluate the amount of variance in our outcome variables on the classroom level. For some of the outcomes, the ICC(1) coefficients were significant; that is, the levels of students in the same classroom were not independent of each other. Those outcomes were teacher-rated achievement in teacher-centered learning, ICC(1) = 0.10, *F*(17, 237) = 2.644, *p* < 0.001; teacher-rated achievement in group work, ICC(1) = 0.11, *F*(17, 237) = 2.687, *p* < 0.001; self-rated achievement in teacher-centered learning, ICC(1) = 0.05, *F*(17, 237) = 1.757, *p* = 0.035; flow in individual tasks, ICC(1) = 0.08, *F*(17, 237) = 2.331, *p* = 0.003; and enjoyment of group work, ICC(1) = 0.08, *F*(17, 237) = 2.150, *p* = 0.006. Based on this non-independence, we decided to run multilevel analyses to address the study’s research questions.

We ran random-intercept models using the lme4 package (Bates et al., 2015) in R (R Core Team, 2013), that is, the respective intercepts could vary between the classrooms. Adding a random slope to the models did not yield an increase in explained variance; hence, we report the results of the random-intercept models. The models used restricted maximum likelihood (REML) estimation. We used lmerTest (Kuznetsova et al., 2017) to compute *p*-values. In the main analyses, we applied an alpha level of α = 0.01 to account for the effects of multiple testing. Given the associations of various study variables with age, gender, and ability-based school track (vocational or academic orientation; see Tables 1, 2), we decided to include these variables as covariates in the analyses testing the hypotheses.

**TABLE 2:** Descriptive statistics, correlations with age, gender, and school track, and intercorrelations for intelligence and dependent variables.

	Descriptives					Intercorrelations											
	*M*	*SD*	*r*_*age*_	*r*_*gender*_	*r*_*track*_	(2)	(3)	(4)	(5)	(6)	(7)	(8)	(9)	(10)	(11)	(12)	(13)
(1) Intelligence	100.51	8.73	0.05	–0.05	0.56	0.17	0.18	0.28	0.17	0.06	0.13	0.12	0.22	0.04	–0.09	0.12	0.02
(2) Teacher-rated achievement: teacher-centered	5.02	1.22	–0.06	0.02	0.02		0.43	0.35	0.29	0.26	0.21	0.21	0.27	0.16	0.14	0.13	0.07
(3) Teacher-rated achievement: individual tasks	5.19	1.24	–0.16	0.24	–0.04			0.51	0.16	0.33	0.14	0.20	0.27	0.11	0.07	0.29	–0.02
(4) Teacher-rated achievement: group work	4.94	1.36	–0.11	0.34	0.11				0.17	0.13	0.22	0.16	0.17	0.15	0.02	0.18	0.09
(5) Self-rated achievement: teacher-centered	4.96	1.20	–0.12	0.00	–0.06					0.40	0.40	0.60	0.45	0.34	0.54	0.12	0.15
(6) Self-rated achievement: individual tasks	5.50	1.08	–0.04	0.09	0.06						0.32	0.40	0.64	0.24	0.13	0.56	0.03
(7) Self-rated achievement: group work	5.38	1.07	–0.04	0.13	–0.10							0.34	0.29	0.49	0.23	0.03	0.60
(8) Flow: teacher-centered	4.38	0.91	–0.14	0.01	0.03								0.65	0.57	0.42	0.17	0.17
(9) Flow: individual tasks	4.70	1.05	–0.16	0.00	0.16									0.50	0.21	0.39	–0.01
(10) Flow: group work	4.64	0.98	–0.15	0.05	0.05										0.24	0.12	0.42
(11) Enjoyment: teacher-centered	4.58	1.64	–0.08	–0.02	–0.08											0.03	0.17
(12) Enjoyment: individual tasks	4.81	1.59	–0.05	0.17	0.11												–0.27
(13) Enjoyment: group work	5.84	1.34	–0.05	0.00	–0.04												

**N = 255. Intelligence: Age-standardized scores (M = 100, SD = 10). Flow, achievement, and enjoyment: Range 1–7. Age: 12.42–18.75 years. Gender: 0 = male, 1 = female. Track: 0 = school with vocational orientation, 1 = school with academic orientation. Correlations r ≥ 0.13 are significant at p < 0.05.**

## Results

### Descriptive Statistics

Descriptive statistics for character strengths and correlations with age, gender, and school track (vocational or academic orientation) are shown in Table 1.

As displayed in Table 1, some small- and medium-sized correlations with demographic variables emerged. Descriptive statistics of intelligence and the dependent variables (school achievement, flow experience, and enjoyment in three learning situations), as well as the respective intercorrelations are displayed in Table 2.

Intelligence was positively related to achievement in all three situations (with the exception of self-rated achievement in individual tasks) and to flow experience in individual tasks, but unrelated to the remaining outcome variables. Both achievement and flow ratings showed high intercorrelations between the three situations, but also seemed separable. Enjoyment ratings seemed to overlap less between the situations, with the enjoyment of individual tasks being negatively related to the enjoyment of group work. The results also show generally small to medium-sized positive correlations between achievement and flow as well as between achievement and enjoyment and medium to large correlations between flow and enjoyment. With the exception of achievement in and enjoyment of group work, the outcomes regarding one type of situation were always positively related.

### Multilevel Analyses

The main analyses refer to the relationships between character strengths and outcomes (teacher- and self-rated achievement, flow, and enjoyment) while controlling for age, gender, school track, and intelligence. The results of the analyses regarding achievement are displayed in Table 3, the results without a control for intelligence are displayed in Supplementary Table S1.

**TABLE 3:** Fixed effects (standardized) of intelligence and character strengths predicting self- and teacher-rated school achievement in three learning situations (controlling for influences of age, gender, school track, and for character strengths also for intelligence).

	Teacher-rated achievement			Self-rated achievement		
	Teacher-centered learning	Individual tasks	Group work	Teacher-centered learning	Individual tasks	Group work
Intelligence	0.23*	0.30*	0.35*	0.20*	0.08	0.17
**Character strengths**						
Creativity	–0.07	–0.06	0.04	0.21*	0.21*	0.21*
Curiosity	0.10	0.13	0.11	0.18*	0.19*	0.09
Judgment	0.07	0.04	0.11	0.20*	0.26*	0.15*
Love of learning	0.19*	0.16*	0.13	0.35*	0.42*	0.16*
Perspective	0.13	0.02	0.15*	0.25*	0.24*	0.24*
Bravery	0.18*	0.00	0.10	0.20*	0.19*	0.07
Perseverance	0.22*	0.12	0.14	0.32*	0.34*	0.21*
Honesty	0.16*	0.05	0.05	0.11	0.24*	0.19
Zest	0.26*	0.03	0.13	0.37*	0.25*	0.16*
Love	0.13	–0.02	0.09	0.28*	0.14	0.09
Kindness	0.12	0.02	0.13	0.12	0.12	0.14
Social intelligence	0.10	–0.01	0.10	0.19*	0.24*	0.19*
Teamwork	0.20*	0.03	0.17*	0.12	0.26*	0.41*
Fairness	0.16*	0.14	0.13	0.06	0.25*	0.19*
Leadership	0.13	–0.09	0.06	0.25*	0.11	0.25*
Forgiveness	0.12	0.10	0.06	0.07	0.17*	0.20*
Humility	0.03	0.04	0.03	–0.11	0.15*	0.12
Prudence	0.07	0.11	0.15*	0.10	0.22*	0.09
Self-regulation	0.14	0.08	0.04	0.11	0.34*	0.18*
Beauty	–0.04	–0.05	0.10	0.20*	0.13	0.11
Gratitude	0.19*	0.04	0.11	0.23*	0.20*	0.17
Hope	0.20*	0.07	0.10	0.34*	0.25*	0.15
Humor	0.01	–0.12	0.06	0.07	0.00	–0.03
Spirituality	0.04	0.02	0.05	0.09	0.13	0.05

N = 255. Beauty = Appreciation of beauty and excellence. * p < 0.01 (one-tailed).

As shown in Table 3, in line with our expectations, and across both self- and teacher-ratings love of learning, perseverance, zest, and hope were positively related to achievement in teacher-centered learning, and love of learning was also positively related to achievement in individual tasks. However, we did not find the expected association between self-regulation and achievement in teacher-centered learning and the associations of perseverance and self-regulation with achievement in individual tasks were only found in self-ratings of achievement. With regards to achievement in group work, the hypothesized positive relations with perspective and teamwork were found across both ratings. In contrast, no significant relationships for love and kindness were observed and the character strengths of judgment, love of learning, zest, social intelligence, fairness, and leadership were only associated with self-rated achievement in group work. Additionally, we found several strengths to positively relate to teacher-rated achievement in teacher-centered learning (i.e., bravery, honesty, fairness, teamwork, and gratitude) and in group work (i.e., prudence), as well as a larger number of strengths to positively relate to self-rated achievement.

Considering flow experiences, we found that, as expected, the strengths of creativity, judgment, love of learning, perseverance, zest, self-regulation, and hope were positively related to flow across the different learning situations beyond intelligence (see Table 4 and Supplementary Table S2 for results without control for intelligence). Curiosity did not show the expected positive relationships with flow experiences. Perspective, love, social intelligence, teamwork, fairness, and leadership (but not kindness) were also additionally related with flow in group work.

**TABLE 4:** Fixed effects (standardized) of intelligence and character strengths predicting flow and enjoyment in three learning situations (controlling for influences of age, gender, school track, and for character strengths also for intelligence).

	Flow			Enjoyment		
	Teacher-centered learning	Individual tasks	Group work	Teacher-centered learning	Individual tasks	Group work
Intelligence	0.13	0.16	0.00	–0.07	0.07	0.06
**Character strengths**						
Creativity	0.23*	0.24*	0.21*	0.16	0.19*	0.08
Curiosity	0.13	0.14	0.11	0.17*	0.20*	–0.04
Judgment	0.28*	0.31*	0.21*	0.16*	0.23*	0.02
Love of learning	0.34*	0.40*	0.18*	0.26*	0.35*	–0.09
Perspective	0.21*	0.29*	0.21*	0.25*	0.08	0.10
Bravery	0.12	0.20*	0.05	0.11	0.03	–0.03
Perseverance	0.35*	0.41*	0.22*	0.23*	0.11	–0.02
Honesty	0.16*	0.24*	0.14	0.09	0.05	0.09
Zest	0.32*	0.26*	0.20*	0.18*	0.10	0.04
Love	0.22*	0.20*	0.17*	0.14	–0.03	0.14
Kindness	0.15	0.08	0.12	0.03	–0.01	0.10
Social intelligence	0.25*	0.26*	0.22*	0.14	0.08	0.09
Teamwork	0.16*	0.16*	0.25*	0.04	0.04	0.31*
Fairness	0.15	0.28*	0.17*	0.05	0.20*	0.02
Leadership	0.14	0.16*	0.20*	0.14	–0.01	0.12
Forgiveness	0.10	0.15*	0.13	0.07	0.06	0.11
Humility	0.01	0.13	0.07	–0.13	0.13	0.09
Prudence	0.26*	0.28*	0.23*	0.14	0.14	0.03
Self-regulation	0.20*	0.28*	0.18*	0.01	0.19*	0.04
Beauty	0.16*	0.17*	0.17*	0.13	0.20*	0.02
Gratitude	0.14	0.16*	0.11	0.08	0.10	0.14
Hope	0.30*	0.34*	0.16*	0.20*	0.14	0.00
Humor	–0.04	–0.02	0.00	0.04	–0.06	0.03
Spirituality	0.06	0.11	0.01	0.10	0.04	0.01

N = 255. Beauty = Appreciation of beauty and excellence. * p < 0.01 (one-tailed).

In line with our expectations, love of learning, perseverance, zest, and hope were associated with enjoying teacher-centered learning, whereas no relationships were found with self-regulation (see Table 4). Love of learning and self-regulation (but not perseverance) were predictors of enjoying individual tasks, and only the character strength of teamwork predicted enjoying group work. In addition, enjoying teacher-centered learning was also positively related to curiosity, judgment, and perspective and enjoying individual tasks was also positively related to creativity, curiosity, judgment, fairness, and appreciation of beauty and excellence.

## Discussion

**FIGURE 2:**
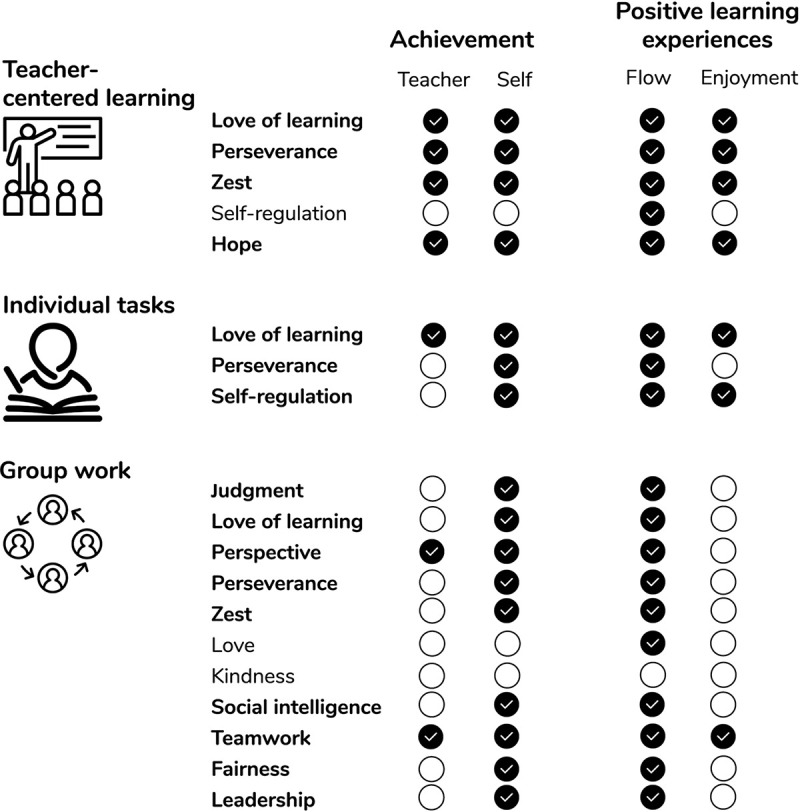
Overview of results in relation to hypotheses. Boldface = positive relationships with at least one indicator of achievement and one indicator of positive learning experiences in the respective learning situation.

The present study followed the principles of trait activation theory in testing the extent to which character strengths show differential trait-outcome relationships across different learning situations that are assumed to activate different sets of character strengths. In doing so, it demonstrated differential relationships of positively valued traits with both achievement and positive learning experiences (flow and enjoyment) across different learning situations beyond cognitive ability. The results are summarized in Figure 2, which gives an overview on the hypotheses supported and not supported by the observed results.

With regard to achievement in different learning situations, we found support for both the idea that certain strengths (such as love of learning and perseverance) are conducive to school achievement in general and the idea that other strengths are activated and contribute to achievement only in specific learning situations.

For instance, the character strength of zest was found to be of particular relevance for achievement and positive learning experiences in *teacher-centered learning*. In this learning situation, students seem to be mostly required to keep up a level of focus and activity, which is favored by approaching the situation with zest. Previous research has demonstrated that extraversion tends to show no (or even negative) relationships with overall academic achievement, at least in secondary and tertiary education (Poropat, 2009). Nonetheless, studies using specific performance criteria, such as oral participation in class (Furnham and Medhurst, 1995), report a positive relationship of extraversion with these achievement criteria, arguably because extraverted behaviors help interact with teachers. The character strength of zest might capture some of the most relevant aspects of extraversion’s facet “activity” that contribute to an advantage in interacting with teachers in teacher-centered learning. Additionally, the character strength of hope was positively related to all four outcome measures regarding teacher-centered learning, in line with expectations. Hope has been shown to be predictive of academic achievement in a variety of educational settings (e.g., Day et al., 2010; Gallagher et al., 2017) and the present results suggest that these relationships found with overall GPA may in part be driven by teacher-centered learning situations, in which hope seems to be particularly activated.

Achievement in *individual tasks* seems to be least explained by character strengths, which might be because it relates least to overt behavior and is thus more difficult to be rated from the teacher’s perspective. Nonetheless, we also found some evidence for the expected relevance of self-regulation, though only with regard to self-reported measures. However, no relationships were found for self-regulation with achievement in *teacher-centered learning*. As self-regulation is a relatively common individual difference variable studied in relation to academic achievement (for an overview, see, e.g., Duckworth and Carlson, 2013), the notion of differential trait-outcome relationships for different learning situations might also be relevant for this research.

In line with our expectations, the character strengths of perspective and teamwork were positively related to both teacher- and self-rated achievement in *group work*. Previous research (Kappe and van der Flier, 2010) investigating the personality dimensions of the five-factor model was not able to find the expected relationships between agreeableness and performance in a learning situation involving a group project. Thus, the narrower traits of character strengths, and traits such as teamwork in particular, might be better suited than the broader and “neutral” dimension of agreeableness to describe individual differences relevant to doing well in a task completed in a group. However, the character strengths of love and kindness were unrelated to both teacher- and self-rated achievement in group work. Both strengths have been found to be of particular relevance for positive peer relationships in the classroom (Wagner, 2019; Wagner and Ruch, 2020), but it seems that this advantage does not necessarily extend into improved performance in situations that require cooperation with peers.

The present study also showed that specific traits can offer a deeper understanding of relationships with outcomes than broader traits. For example, Kappe and van der Flier (2010) found openness to experience to relate to lower performance ratings in group settings and argued that bringing a lot of different perspectives into the discussion can distract from completing a group task in a timely manner. However, the strength of judgment covers exactly this specific aspect (i.e., considering different perspectives), whereas openness to experience is a much broader and non-valued trait that includes many different aspects, which might also be relevant to how openness to experience contributes to performing in a group task. Our results suggest that the narrower strength of judgment is conducive to self-rated achievement and to flow experiences in group settings, at least in the context of secondary school. Thus, specific traits allow for a more nuanced examination of the relationships between personality traits and educational outcomes.

When we assess the full picture of relationships with achievement against previous studies on the role of character strengths for overall school achievement (e.g., Wagner and Ruch, 2015), we find that the strengths of love of learning and perseverance show the strongest and most consistent relationships with achievement across various learning situations beyond the influence of cognitive ability. Wagner and Ruch (2015) found that, in addition to love of learning and perseverance, overall school achievement was positively correlated with zest, prudence, gratitude, hope, and perspective across two samples. In the present study, zest, hope, and perspective show at least some evidence of differential trait-outcome relationships, with zest and hope, in particular, being mostly related to performance in teacher-centered learning. There were no hypotheses for gratitude and prudence; however, gratitude was linked with both teacher- and self-rated achievement, but not with positive learning experiences, in teacher-centered learning, and prudence demonstrated a positive relationship with teacher-rated achievement in group tasks. Thus, the present results offer some support that these character strengths are predictive of academic achievement even when controlling for the influence of cognitive ability.

With regard to flow experiences in the different learning situations, we also found support for our expectations. At the same time, while some character strengths showed differential patterns of relationships (such as love of learning, which was associated more strongly with flow in individual than in group tasks, or self-regulation, which showed the strongest association with flow in individual tasks), many others showed similar associations across the different learning situations. This might suggest that certain traits are generally linked to a proneness to experience flow in the school setting, irrespective of the learning situation. A number of strengths might generally predispose students to enter a flow state in the educational setting (such as creativity, judgment, and love of learning). In contrast, other strengths can be assumed to be conducive to entering a flow state (such as zest or hope) or staying in a flow state in the face of distractions (such as perseverance or self-regulation; see Wagner et al., 2020; Wagner and Ruch, 2020). Future research would benefit from a more fine-grained analysis of situations in which flow occurs at school to allow uncovering differential associations with personality traits.

Finally, when considering enjoyment of the three learning situations, the relationships varied a lot between the different learning situations; that is, results were much more in line with the notion of different character strengths predisposing individuals to enjoy learning in different contexts. These findings are again in line with the arguments of trait activation theory, which also assumes that the display of traits leads to satisfaction. Specifically, if a contextual cue activates a trait and the trait is displayed, the individual will in turn be likely to enjoy this situation.

In our analyses, we controlled for intelligence with the aim to study the incremental contribution of character strengths in predicting educational outcomes beyond cognitive ability. In theory, character strengths and intelligence do not overlap, and also the observed overlap in the present study was small. It should be considered, though, that we used a comprehensive measure of cognitive ability that includes both fluid and crystallized aspects of intelligence. Character strengths demonstrated incremental validity even above this broadly defined assessment of intelligence, suggesting that they represent useful constructs to study relationships between narrower traits and achievement as well as positive experiences at school (see O’Connor and Paunonen, 2007). The size of the relationships for intelligence and the relevant character strengths with the main outcome (teacher-rated achievement) was overall comparable. In the case of teacher-rated achievement in teacher-centered learning, when intelligence was considered together with love of learning, perseverance, zest, teamwork, or hope, the relationship proved to be numerically smaller yet very similar-sized. For the other two learning situations, the relationships of achievement with intelligence were somewhat stronger than the associations of the relevant strengths with achievement, albeit also of comparable size. These analyses include three different methods (intelligence test, self-reported character strengths, and teacher-rated achievement) and intelligence was measured more reliably than character strengths. As a consequence, the findings represent a strong argument for the relevance of positively valued traits, such as character strengths, in predicting achievement in the educational context. With regard to self-rated achievement, flow, and enjoyment in the three learning situations, character strengths clearly outperform intelligence in their predictive power.

Our findings contribute to the understanding of specific contextual factors that determine how personality traits relate to educational outcomes. Learning situations that vary with regards to demands, type, and amount of social interaction should be further considered as contextual factors in understanding these complex relationships. Future research should also study whether strengths-related behavior varies as expected between the different learning situations. The three learning situations we studied only represent one of many aspects in which achievement and positive learning experiences can vary; other characteristics, such as the subject content as well as relationships with classmates and teachers involved, might be of equal importance. Nonetheless, performing well in different types of social interactions might also be relevant in later life, such as in university education or at the workplace. Thus, the present findings might also have implications for how character strengths relate to different aspects of performance in adulthood (see Harzer and Ruch, 2014). Furthermore, when considering the possibility of interventions to foster certain personality traits or character strengths, information on the role of specific contexts, such as learning situations, should be considered. Another practical recommendation following the current findings could extend to designing schools and planning specific lessons. Based on the present results, offering a variation or a choice of learning situations would allow different strengths to be activated and as a consequence, more students (with diverse strengths) to be able to perform well and enjoy learning.

### Strengths and Limitations

The present study has several strengths. For instance, it uses different data sources (self-reports, standardized tests, teacher ratings) and different time points (3 months apart) to reduce or eliminate the influence of common method bias. However, the present results also need to be interpreted in light of several limitations. First, the learning situations selected in the present study certainly do not cover all situations that are potentially relevant to learning in a classroom, and the descriptions provided were rather general. Thus, students and teachers might have differed in their understanding of the types of situations described. Second, teachers might not be the best informants about achievement in group work; hence, future studies might also consider peer ratings. Third, the assessment of all outcomes relied on ratings of habitual behavior (teacher- and self-rated school achievement) or habitual experiences (self-reported flow experience and enjoyment). In future studies, it would be desirable to assess these outcomes through either observation or experience-sampling methods. Fourth, even though participants were diverse to some extent (attending different school tracks in several communities in German-speaking Switzerland), the present results might not extend to other cultural contexts. Finally, an important limitation is that it is impossible to draw conclusions regarding directionality or causality based on the present results.

## Conclusion

The present study looked at the role of students’ character strengths in predicting educational outcomes beyond the influence of cognitive ability. Specifically, we asked the question: Which students perform well and have positive experiences in different situations at school, irrespective of their intelligence? We focused on three learning situations and the results demonstrated that the associations differed between those situations. Our results support the notion that character strengths represent a useful framework for a nuanced examination of the complex relationships between personality traits and educational outcomes. Overall, quite a large number of character strengths are relevant when predicting different educational outcomes and the strengths’ narrow definitions allow for depicting differential relationships.

## Data Availability Statement

The datasets generated for this study are available on request to the corresponding author.

## Ethics Statement

The studies involving human participants were reviewed and approved by the University of Zurich, Zurich, Switzerland; Ethikkommission (für psychologische und verwandte Forschung). All participants gave their written consent and participated voluntarily. Students under the age of 14 years were provided written permission to participate by a parent or legal guardian.

## Author Contributions

LW and WR contributed to the conception and design of the study. MH and HW collected the data and wrote sections of the manuscript. LW performed the statistical analysis and wrote the first draft of the manuscript. All authors contributed to the article and approved the submitted version.

## Conflict of Interest

WR is a senior scientist at the VIA Institute on Character, which holds the copyright of the VIA-Youth. The remaining authors declare that the research was conducted in the absence of any commercial or financial relationships that could be construed as a potential conflict of interest.
