# Supplementary material for: Character Strengths Are Related to Students’ Achievement, Flow Experiences, and Enjoyment in Teacher-Centered Learning, Individual, and Group Work Beyond Cognitive Ability
Source: Front Psychol. 2020 Jul 16;11:1324. doi: 10.3389/fpsyg.2020.01324 (PMC7378955; doi:10.3389/fpsyg.2020.01324)
Supplement: Supplementary file 1 [file Table_1.docx]

Table S1

Fixed Effects (Standardized) of Character Strengths Predicting Self- and Teacher-Rated School Achievement in Three Learning Situations (Controlling for Influences of Age, Gender, and School Track)

|  | Teacher-rated achievement | | | Self-rated achievement | | |
| --- | --- | --- | --- | --- | --- | --- |
|  | Teacher-centered learning | Individual tasks | Group work | Teacher-centered learning | Individual tasks | Group work |
| Creativity | -.06 | -.04 | .06 | .22* | .21* | .22* |
| Curiosity | .13 | .17* | .15* | .21* | .19* | .11 |
| Judgment | .07 | .04 | .10 | .20* | .26* | .15* |
| Love of learning | .21* | .20* | .18* | .36 | .42* | .18* |
| Perspective | .15* | .05 | .19* | .26* | .25* | .25* |
| Bravery | .20* | .02 | .12 | .21* | .20* | .08 |
| Perseverance | .24* | .15* | .17* | .34* | .35* | .22* |
| Honesty | .17* | .07 | .07 | .12 | .25* | .20* |
| Zest | .28* | .07 | .17* | .39* | .25* | .18* |
| Love | .13 | -.02 | .09 | .29* | .14 | .09 |
| Kindness | .15 | .05 | .16* | .14 | .12 | .15* |
| Social intelligence | .11 | .00 | .11 | .19* | .24* | .19* |
| Teamwork | .23* | .08 | .22* | .14 | .26* | .42* |
| Fairness | .17* | .15* | .15* | .07 | .25* | .20* |
| Leadership | .15* | -.05 | .10 | .27* | .12 | .27* |
| Forgiveness | .14 | .13 | .09 | .08 | .18* | .21* |
| Humility | .04 | .05 | .04 | -.11 | .15* | .12 |
| Prudence | .06 | .09 | .13 | .09 | .21* | .08 |
| Self-regulation | .15* | .10 | .06 | .12 | .34* | .18* |
| *(Table S1 continues)* | | | | | | |
|  | Teacher-rated achievement | | | Self-rated achievement | | |
|  | Teacher-centered learning | Individual tasks | Group work | Teacher-centered learning | Individual tasks | Group work |
| Beauty | -.02 | .00 | .14 | .22* | .14 | .13 |
| Gratitude | .20* | .06 | .13 | .25* | .21* | .17* |
| Hope | .22* | .10 | .13 | .35* | .26* | .17* |
| Humor | .02 | -.11 | .07 | .07 | .00 | -.03 |
| Spirituality | .05 | .02 | .07 | .10 | .13 | .05 |

*Note.* *N* = 255. Beauty = Appreciation of beauty and excellence.

* *p* < .01 (one-tailed)
