# Supplementary material for: Character Strengths Are Related to Students’ Achievement, Flow Experiences, and Enjoyment in Teacher-Centered Learning, Individual, and Group Work Beyond Cognitive Ability
Source: Front Psychol. 2020 Jul 16;11:1324. doi: 10.3389/fpsyg.2020.01324 (PMC7378955; doi:10.3389/fpsyg.2020.01324)
Supplement: Supplementary file 2 [file Table_2.docx]

Table S2

Fixed Effects (Standardized) of Character Strengths Predicting Flow and Enjoyment in Three Learning Situations
(Controlling for Influences of Age, Gender, and School Track)

|  | Flow | | | Enjoyment | | |
| --- | --- | --- | --- | --- | --- | --- |
|  | Teacher-centered learning | Individual tasks | Group work | Teacher-centered learning | Individual tasks | Group work |
| Creativity | .24* | .25* | .21* | .15* | .20* | .09 |
| Curiosity | .15* | .16* | .11 | .15* | .20* | -.03 |
| Judgment | .28* | .31* | .21* | .16* | .23* | .02 |
| Love of learning | .35* | .41* | .17* | .24* | .35* | -.08 |
| Perspective | .22* | .31* | .20* | .24* | .08 | .11 |
| Bravery | .13 | .21* | .05 | .10 | .03 | -.02 |
| Perseverance | .36* | .42* | .22* | .22* | .12 | -.02 |
| Honesty | .16* | .25* | .14 | .09 | .06 | .09 |
| Zest | .33* | .27* | .19* | .17* | .10 | .04 |
| Love | .23* | .20* | .17* | .14 | -.03 | .14 |
| Kindness | .16 | .10 | .12 | .02 | .00 | .10 |
| Social intelligence | .25* | .26* | .22* | .14 | .08 | .10 |
| Teamwork | .18* | .18* | .24* | .03 | .05 | .30* |
| Fairness | .16* | .29* | .17* | .05 | .20* | .02 |
| Leadership | .15* | .18* | .19* | .13 | .00 | .13 |
| Forgiveness | .11 | .16* | .13 | .07 | .07 | .12 |
| Humility | .01 | .13 | .07 | -.13 | .13 | .09 |
| Prudence | .25* | .26* | .23* | .15* | .13 | .03 |
| Self-regulation | .21* | .29* | .18* | .00 | .20* | .04 |
| *(Table S2 continues)* | | | | | | |
|  | Flow | | | Enjoyment | | |
|  | Teacher-centered learning | Individual tasks | Group work | Teacher-centered learning | Individual tasks | Group work |
| Beauty | .18* | .19* | .16* | .12 | .21* | .03 |
| Gratitude | .15* | .17* | .11 | .08 | .11 | .15* |
| Hope | .31* | .35* | .15* | .19* | .14 | .01 |
| Humor | -.04 | -.01 | .00 | .04 | -.06 | .03 |
| Spirituality | .06 | .12 | .01 | .09 | .04 | .01 |

Note. *N* = 255. Beauty = Appreciation of beauty and excellence.

* *p* < .01 (one-tailed)
