# Supplementary material for: Character Strengths Are Related to Students’ Achievement, Flow Experiences, and Enjoyment in Teacher-Centered Learning, Individual, and Group Work Beyond Cognitive Ability
Source: Front Psychol. 2020 Jul 16;11:1324. doi: 10.3389/fpsyg.2020.01324 (PMC7378955; doi:10.3389/fpsyg.2020.01324)
Supplement: Supplementary file 3 [file Presentation_1.pdf]

Supplementary materials for „Character Strengths are Related to Students' Achievement, Flow Experiences, and Enjoyment in Teacher-Centered Learning, Individual, and Group Work Beyond Cognitive Ability“

(1) Descriptions used for teacher-ratings

German descriptions:

Frontalunterricht: In der Schule gibt es Situationen in denen die Lehrperson vor der Klasse steht und etwas erklärt. Diese Situationen bezeichnen wir als "Frontalunterricht".

Einzelarbeit: In der Schule gibt es Situationen in denen die Lehrperson einen Auftrag erteilt hat. In einigen dieser Situationen sollen die Schülerinnen und Schüler die Aufgabe alleine bearbeiten. Diese Situationen bezeichnen wir als "Einzelarbeit".

Gruppenarbeit: In der Schule gibt es Situationen in denen die Lehrperson einen Auftrag erteilt hat. In einigen dieser Situationen sollen die Schülerinnen und Schüler die Aufgabe in Kleingruppen von zwei bis fünf Personen bearbeiten. Diese Situationen bezeichnen wir als "Gruppenarbeit".

Tentative English translations:

Teacher-centered learning: At school, there are situations, in which the teacher is in front of the class and explains at subject matter. We refer to these situations as “teacher-centered learning”.

Individual tasks: At school, there are situations, in which the teacher gives the students a task to complete. In some of these situations, students are asked to work on these tasks individually. We refer to these situations as “individual tasks”.

Group work: At school, there are situations, in which the teacher gives the students a task to complete. In some of these situations, students are asked to work on these tasks in groups of two to five students. We refer to these situations as “group work”.

(2) Descriptions used for students‘ self-ratings

German descriptions:

Frontalunterricht: In der Schule gibt es Situationen in denen die Lehrperson vor der Klasse steht und etwas erklärt. Diese Situationen bezeichnen wir als "Frontalunterricht".

Einzelarbeit: In der Schule gibt es Situationen, in denen die Lehrperson einen Auftrag erteilt hat. In einigen dieser Situationen sollst du die Aufgabe alleine bearbeiten. Diese Situationen bezeichnen wir als "Einzelarbeit".

Gruppenarbeit: In der Schule gibt es Situationen, in denen die Lehrperson einen Auftrag erteilt hat. In einigen dieser Situationen sollst du die Aufgabe in Kleingruppen mit ein bis vier deiner Mitschüler/innen bearbeiten. Diese Situationen bezeichnen wir als „Gruppenarbeit“.

Tentative English translations:

Teacher-centered learning: At school, there are situations, in which the teacher is in front of the class and explains at subject matter. We refer to these situations as “teacher-centered learning”.

Individual tasks: At school, there are situations, in which the teacher gives you a task to complete. In some of these situations, you are asked to work on these tasks individually. We refer to these situations as “individual tasks”.

Group work: At school, there are situations, in which the teacher you a task to complete. In some of these situations, you are asked to work on these tasks in a group together with one to four of your classmates. We refer to these situations as “group work”.
